# Association of neutrophil-to-prognostic nutritional index ratio with long-term mortality in acute myocardial infarction from Chinese and US cohorts

**DOI:** 10.3389/fnut.2026.1814469

**Published:** 2026-06-18

**Authors:** Hongqiang Li, Jiachen Luo, Gang Li, Fei Yang, Zhenhua Wang, Yidong Wei

**Affiliations:** 1 General ICU, The First Affiliated Hospital of Zhengzhou University, Zhengzhou, China; 2 Department of Cardiology, Shanghai Tenth People's Hospital, Tongji University, Shanghai, China; 3 Department of Cardiology, The First Affiliated Hospital of Zhengzhou University, Zhengzhou, China

**Keywords:** acute myocardial infarction, inflammation, mortality, neutrophils, prognosis, prognostic nutritional index

## Abstract

**Background:**

Despite advancements in reperfusion strategies, patients with acute myocardial infarction (AMI) face significant risks of long-term mortality. Systemic inflammation and nutritional/immune depletion synergistically impact prognosis. This study investigated the prognostic value of the neutrophil count to prognostic nutritional index ratio (NPNR)—a novel composite index reflecting inflammatory burden and nutritional reserves—in AMI patients.

**Methods:**

This dual-cohort study included 2020 patients from the NOAFCAMI-SH registry in China and 1,433 patients from the MIMIC-IV database in the USA. The primary endpoint was all-cause mortality; the secondary endpoint was cardiovascular mortality. Associations were analyzed using Kaplan-Meier curves, Cox regression, and restricted cubic splines.

**Results:**

During a median 2.5-year follow-up in the discovery cohort, 285 all-cause and 234 cardiovascular deaths occurred. Higher NPNR levels correlated with myocardial injury and lower LVEF. Kaplan-Meier analysis showed significantly worse survival with elevated NPNR (*P* < 0.001). In fully adjusted models, NPNR independently predicted all-cause (HR: 2.59, 95% CI: 1.21–5.54) and cardiovascular mortality (HR: 2.87, 95% CI: 1.34–6.15). Compared to the lowest tertile, patients in the highest NPNR tertile had a 2.19-fold higher risk of all-cause mortality (HR: 2.19, 95% CI: 1.55–3.09) and a 2.36-fold higher risk of cardiac death (HR: 2.36, 95% CI: 1.60–3.48).

This was validated in the MIMIC-IV cohort (HR:1.91 for 1-year mortality). The association was particularly strong in patients aged ≥65 years. Adding NPNR to a baseline clinical model significantly improved overall discrimination (C-statistic: 0.807–0.811, *P* = 0.011) and risk reclassification for mortality (continuous NRI = 0.368, IDI = 0.011; both *P* = 0.013).

**Conclusion:**

NPNR is a robust, independent predictor of long-term all-cause and cardiac mortality in AMI patients, validated in the external MIMIC-IV cohort. This simple biomarker effectively aids in risk stratification.

## Introduction

Acute myocardial infarction (AMI) remains a leading cause of death worldwide and a significant contributor to long-term cardiovascular disability (1). Despite considerable advancements in reperfusion strategies, particularly percutaneous coronary intervention (PCI), and the implementation of evidence-based pharmacotherapy, the residual risk of adverse events following AMI remains substantial (2, 3). Patient outcomes demonstrate significant variability, highlighting the urgent need for enhanced prognostic strategies for detecting vulnerable patients requiring aggressive therapeutic interventions (4).

The pathophysiological landscape of AMI extends beyond the acute thrombotic event, encompassing a profound systemic inflammatory response (5, 6). Neutrophils play a crucial role as early effectors, contributing to myocardial ischemia-reperfusion injury, adverse ventricular remodeling, and plaque instability (7, 8). Additionally, malnutrition, often underrecognized in cardiovascular patients, is closely associated with frailty, immune dysfunction, and poor prognosis. Traditional indicators such as serum albumin and lymphocyte count serve as reflections of nutritional status and immune competence (9). The pronounced inflammatory state significantly accelerates catabolism, thereby depleting nutritional reserves and establishing a detrimental cycle in which “hyper-inflammation” and “nutritional/immune depletion” mutually exacerbate each other (10).

This intricate interaction underscores the limitations of current unidimensional biomarkers. Existing indices, such as the prognostic nutritional index (PNI) and the neutrophil-to-lymphocyte ratio (NLR), capture only partial aspects of patient vulnerability, evaluating either nutritional-immune competence or inflammatory balance in isolation (11–13). A novel composite index, the neutrophil count to prognostic nutritional index ratio (NPNR), amalgamates the “pro-inflammatory driver” with the “protective reserve”. Recent study emphasizes that intersecting systemic inflammatory cascades and nutritional-metabolic deficits—rather than isolated pathways—are the primary drivers of residual cardiovascular risk (14). Preliminary evidence from studies involving critically ill populations, such as elderly patients with sepsis, indicates that NPNR exhibits a significant V-shaped relationship with mortality, underscoring its potential as a robust prognostic tool (15).

To date, the potential of NPNR as a predictor for long-term all-cause and cardiovascular death in diverse AMI populations remains unexplored. Consequently, this study seeks to assess the relationship between admission NPNR levels and long-term adverse outcomes in a contemporary AMI population. We postulate that higher NPNR levels act as an independent predictor of increased long-term all-cause and cardiovascular mortality, offering a simple yet robust marker for clinical risk assessment.

## Methods

### Study design and ethical statement

**Figure 1:**
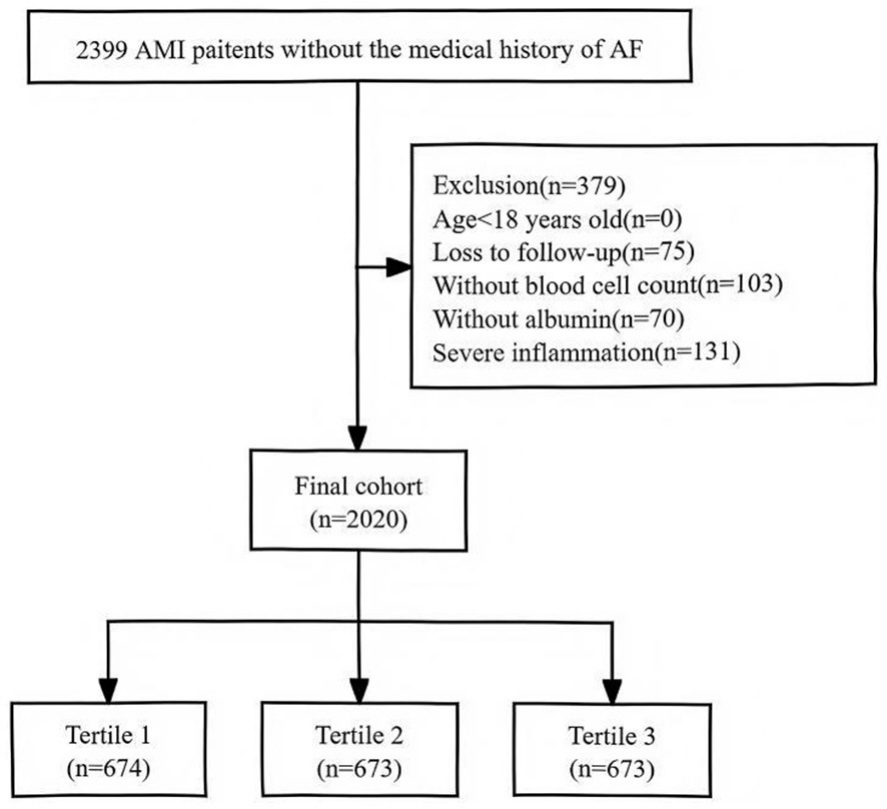
Flowchart of patient selection in the discovery cohort.

This study utilized a dual-cohort design, comprising a local discovery cohort and an external validation cohort, to ensure the robustness and generalizability of our findings. The discovery cohort was derived from the NOAFCAMI-SH registry (ClinicalTrials.gov Identifier: NCT03533543). We initially screened 2,399 patients with AMI admitted to the Cardiac Care Unit (CCU) of Shanghai Tenth People's Hospital between February 2014 and March 2018. AMI cases were identified strictly adhering to the criteria outlined in the Fourth Universal Definition of Myocardial Infarction (16). In accordance with the standardized operating procedures of the NOAFCAMI-SH registry and as previously validated in related studies (17), patients were excluded based on the following criteria: (1) age < 18 years; (2) missing data on admission neutrophil, lymphocyte, or albumin levels; (3) presence of a severe inflammatory response, defined as a white blood cell count ≥ 20 × 10^9^/L or C-reactive protein ≥ 200 mg/L, to minimize confounding effects; and (4) incomplete follow-up data. The final dataset consisted of 2020 patients, selected after rigorous application of the inclusion and exclusion criteria. A detailed patient screening flowchart is provided in Figure 1.

For external validation, we utilized data from the MIMIC-IV database, a comprehensive public repository housing anonymized records of ICU admissions at Beth Israel Deaconess Medical Center from 2008 to 2019. The investigator, Hongqiang Li (Record ID: 64664341), obtained credentialed access to the dataset. We initially screened 7,151 AMI patients identified by International Classification of Diseases (ICD)-9 and ICD-10 codes. To ensure comparability with the discovery cohort and data independence, the following exclusion criteria were applied: (1) records other than the first ICU admission; (2) age < 18 years; (3) length of stay < 24 hours; (4) history of atrial fibrillation, coronary artery bypass grafting (CABG), rheumatic heart disease (RHD), or hyperthyroidism; (5) missing data on neutrophils, lymphocytes, or albumin; (6) severe inflammatory response (white blood cell count ≥ 20 × 10^9^/L or C-reactive protein ≥ 200 mg/L). Following this screening process, a total of 1,433 patients were ultimately included in the validation cohort. The screening flowchart is detailed in Figure S1.

This research adhered to the ethical standards outlined in the Declaration of Helsinki. Ethical clearance for the NOAFCAMI-SH registry was granted by the Ethics Committee of Shanghai Tenth People's Hospital (No. SHSY-IEC-KY-4.1/18–199/01). Regarding the MIMIC-IV dataset, the Institutional Review Boards at both MIT and BIDMC waived the need for patient consent, citing the retrospective nature and anonymization of the data.

### Data collection and variable definition

Researchers conducted a retrospective collection of baseline data utilizing a standardized electronic medical record. To ensure data comparability, all laboratory indicator units were standardized prior to analysis. These indicators were derived from blood samples taken from the cubital vein within 24 h of the patient's admission. The assays were conducted in the central laboratory of Shanghai Tenth People's Hospital. The primary exposure variable in this study was the neutrophil-to-prognostic nutritional index ratio (NPNR), calculated from the initial laboratory data at admission using the formula: NPNR = neutrophil counts ( × 10^9^/L) / [serum albumin (g/dL) + 5 × lymphocyte counts ( × 10^9^/L)]. Participants in the discovery cohort were categorized into tertiles based on NPNR concentrations: T1 ( ≤ 0.118), T2 (0.118–0.175), and T3 (> 0.175). Likewise, the MIMIC-IV population was divided into three groups with the following cutoffs: T1 ( ≤ 0.164), T2 (0.164–0.270), and T3 (>0.270). The analysis incorporated the following covariates: (1) Demographic and clinical characteristics, including age, sex, current smoking status, admission vital signs (heart rate and systolic blood pressure), and the type of myocardial infarction (STEMI or NSTEMI); (2) Comorbidities, such as hypertension, diabetes, dyslipidemia, chronic kidney disease (CKD), and chronic heart failure (CHF); (3) Laboratory and echocardiographic parameters, encompassing complete blood count (red blood cells and platelets), biochemical markers (creatinine, lipid profile, and C-reactive protein), cardiac biomarkers (troponin T and NT-proBNP), and left ventricular ejection fraction (LVEF) measured within 24 h of admission; and (4) Treatment variables, including the use of PCI during hospitalization and medication usage (aspirin, statins, ACEI/ARB, and beta-blockers).

### Follow-up and outcomes

For the discovery group, clinical outcomes were ascertained through telephonic contact and retrospective chart review. The duration of follow-up was measured as the interval between hospital admission and the first endpoint event or the administrative censoring date in April 2019. Survival status and cause of death were independently assessed by two trained cardiologists through a blinded review of medical records and follow-up logs. Our primary outcome was all-cause mortality, while the secondary outcome focused on cardiovascular mortality. The latter included deaths attributed to specific cardiovascular events such as acute myocardial infarction, heart failure, or malignant arrhythmias. Following standard protocols, we categorized any death as cardiovascular in origin unless a non-cardiovascular cause was explicitly recorded. For the validation cohort (MIMIC-IV), mortality information was directly extracted from the database records. Due to the absence of specific cause-of-death adjudication in the MIMIC database, this cohort was used exclusively to validate 1-year all-cause mortality.

### Statistical analysis

Normally distributed variables were presented as mean ± standard deviation (SD) and compared across groups using one-way analysis of variance (ANOVA). Non-normally distributed variables were presented as median (interquartile range, IQR) and compared using the Kruskal-Wallis test. Categorical variables were stratified according to NPNR tertiles and analyzed using the Chi-square test.

Generalized additive models (GAM) incorporating smoothing curve fitting were utilized to assess the potential associations between the NPNR and both peak cardiac troponin T levels and LVEF. The smoothing curve fitting was performed using the gam function. To mitigate the impact of outliers, NPNR values exceeding the 99th percentile were winsorized. Survival curves were constructed using the Kaplan-Meier method, and differences in survival rates among NPNR groups were evaluated using the Log-rank test. In the final cohort, missing values were observed for LVEF (5.8%), initial serum creatinine (0.6%), and NTproBNP (0.2%), and were imputed using the median. Multicollinearity among covariates in the final models was examined using the variance inflation factor (Table S1). The primary analysis employed Cox proportional hazards regression models to investigate the association between NPNR and adverse outcomes. Hazard ratios (HRs) and 95% confidence intervals (CIs) were calculated to quantify the magnitude of risk. Variable selection was guided by the *P*-value (< 0.05) from univariate analysis (Table S2), clinical relevance, and established evidence of prognostic significance in prior myocardial infarction literature.

Three regression models were developed to systematically adjust for confounding variables. Model 1 was unadjusted; Model 2 included adjustments for age and gender; and Model 3 incorporated adjustments for age, gender, smoking status, medical history (including hypertension, diabetes, CKD, and CHF), STEMI, admission vital signs (heart rate and systolic blood pressure), LVEF, PCI, laboratory parameters (such as creatinine, C-reactive protein, peak troponin T, total cholesterol, and HDL-C), and medication use (including aspirin, ACEI/ARB, beta-blockers, and statins). For the discovery cohort, analyses were conducted separately for all-cause and cardiovascular mortality. In the MIMIC-IV validation cohort, adjustments were made based on data availability to encompass variables such as age, gender, ethnicity, admission heart rate, systolic blood pressure, comorbidities (including hypertension, diabetes, CKD, dyslipidemia, and CHF), laboratory markers (such as creatinine and peak troponin T), PCI, and medication usage (including aspirin, ACEI/ARB, beta-blockers, and statins). We conducted a Cox regression analysis within the validation cohort to primarily assess the risk of all-cause mortality and to verify the consistency in the direction and significance of the hazard ratios. Restricted cubic splines were employed to model the relationship between the NPNR and mortality risk. The Receiver Operating Characteristic (ROC) curve was used to evaluate the predictive value of NPNR and SII for all-cause mortality and cardiovascular mortality. This study constructed a baseline clinical model comprising core variables (age, sex, SBP, LVEF, creatinine, Peak TnT, and STEMI). The model's discriminative power was assessed using Harrell's C-index, with significance testing applied to differences in the C-index. The continuous Net Reclassification Improvement Index (NRI) and Comprehensive Discriminant Improvement Index (IDI) were employed to quantify the efficacy of NPNR in risk reclassification. All statistical analyses were prespecified and performed using Stata 18 and R software (version 4.4.3). A two-sided *P* value < 0.05 was considered statistically significant.

### Subgroup and sensitivity analyses

To evaluate the consistency of the prognostic significance of the NPNR across various clinical profiles, subgroup analyses were conducted within the discovery cohort. Stratification factors included age (< 65 vs. ≥65 years), gender (male vs. female), presence of hypertension, diabetes, CKD, and myocardial infarction phenotype (STEMI vs. NSTEMI). Interaction terms were assessed using likelihood ratio tests to ascertain whether these stratification factors significantly influenced the prognostic impact of NPNR. Additionally, to confirm the robustness of our findings and ensure comparability with the MIMIC-IV cohort, a sensitivity analysis was performed within the discovery cohort. This involved truncating the follow-up period to 1 year and re-evaluating the predictive capacity of NPNR for all-cause mortality risk through multivariable Cox regression analysis.

## Results

### Baseline characteristics of participants

In the discovery cohort of 2020 AMI patients followed for a median of 2.5 years, there were 285 all-cause deaths (14.1%) and 234 cardiovascular deaths (11.6%). Table 1 shows baseline characteristics by NPNR tertiles. Patients in the highest tertile (T3) exhibited a more severe clinical presentation than those in the lowest tertile (T1). This group was characterized by a greater prevalence of STEMI, CHF, and CKD, along with elevated heart rates and reduced systolic blood pressure (*P* < 0.001). T3 also showed higher systemic inflammation and myocardial injury, with elevated white blood cells, neutrophils, CRP, NT-proBNP, and troponin T levels. Additionally, T3 had a lower LVEF (47.1 vs. 52.4%), lower TG levels, and was less likely to receive ACEI/ARB therapy. In the T3 group, in-hospital, all-cause, and cardiovascular mortality rates were significantly higher (*P* < 0.001).

**Table 1:** Baseline characteristics of the study population by NPNR tertiles.

Characteristics	Total (*N* = 2020)	T1 (*N* = 674)	T2 (*N* = 673)	T3 (*N* = 673)	*P*-value
Demographics					
Age, years	65.0 (57.0–76.0)	66.0 (58.0–77.0)	64.0 (57.0–76.0)	65.0 (58.0–75.0)	0.104
Male, *n* (%)	1,534 (75.9)	487 (72.4)	527 (78.3)	520 (77.2)	0.026
Current smoker, *n* (%)	877 (43.4)	268 (39.8)	319 (47.4)	290 (43.0)	0.019
Heart rate, bpm	80.0 (69.0–90.0)	76.0 (67.0–86.0)	80.0 (70.0–9.0)	82.0 (70.0–95.0)	<0.001
SBP, mmHg	137(122–154)	140.0 (125–156)	138 (123–154)	133 (115–150)	<0.001
STEMI	1,229 (60.8)	303 (45.0)	417 (62.0)	509 (75.5)	<0.001
PCI	1,683 (83.3)	545 (81.0)	564 (83.8)	574 (85.2)	0.110
Comorbidities, *n* (%)					
Hypertension	1,285 (63.6)	420 (62.4)	434 (64.5)	431 (63.9)	0.713
Diabetes	768 (38.0)	240 (35.7)	257 (38.2)	271 (40.2)	0.227
Dyslipidemia	535 (26.5)	170 (25.3)	181 (26.9)	184 (27.3)	0.668
CKD	187 (9.3)	46 (6.8)	58 (8.6)	83 (12.3)	0.002
CHF	534 (26.4)	123 (18.3)	173 (25.7)	238 (35.3)	<0.001
Laboratory data					
WBC, × 10^9^/L	9.28 (7.37–11.49)	6.96 (5.80–7.88)	9.18 (8.09–10.41)	12.39 (10.71–14.28)	<0.001
Neutrophil, × 10^9^/L	6.82 (5.06–9.08)	4.48 (3.68–5.17)	6.83 (6.10–7.57)	10.23 (8.92–12.14)	<0.001
Lymphocyte, × 10^9^/L	1.57 (1.13–2.10)	1.80 (1.35–2.33)	1.65 (1.22–2.15)	1.27 (0.93–1.76)	<0.001
CRP, mg/L	5.8 (3.2–19.1)	4.0 (3.2–12.8)	5.3 (3.2–16.9)	9.2 (3.3–36.7)	<0.001
Hemoglobin, g/L	134 (122–145)	132 (120–143)	134 (124–146)	135 (121–146)	0.009
Platelet, × 10^9^/L	202 (166–242)	190 (159–224)	202 (166–241)	214 (177–257)	<0.001
Albumin, g/L	39.0 (36.0–41.0)	39.0 (37.0–42.0)	39.0 (37.0–41.0)	38.0 (35.0–41.0)	<0.001
TC, mmol/L	4.4 (3.8–5.0)	4.4 (3.8–5.0)	4.4 (3.9–5.0)	4.4 (3.7–5.0)	0.982
TG, mmol/L	1.4 (1.1–2.0)	1.5 (1.1–2.1)	1.4 (1.1–2.0)	1.3 (1.0–1.7)	<0.001
HDL, mmol/L	1.0 (0.9–1.2)	1.0 (0.9–1.1)	1.0 (0.8–1.1)	1.0 (0.9–1.2)	0.013
LDL, mmol/L	2.6 (2.2–3.2)	2.6 (2.2–3.2)	2.6 (2.2–3.2)	2.6 (2.1–3.3)	0.681
HBA1C, %	6.0 (5.6–7.0)	6.0 (5.6–6.8)	6.0 (5.6–7.0)	6.0 (5.6–7.0)	0.216
Creatinine, μmol/L	78.9 (67.0–94.8)	78.1 (66.1–91.0)	78.1 (67.4–94.6)	80.6 (68.4–99.5)	0.001
NTproBNP, pg/mL	1,581 (758–3,788)	1,154 (549–2,433)	1,439 (678–3,427)	2,478 (1131–6,515)	<0.001
Peak TnT, ng/mL	3.1 (0.8–8.3)	1.3 (0.4–3.8)	3.1 (1.1–8.1)	6.1 (2.3–10.0)	<0.001
Echocardiography					
LAD	37.0 (34.0–41.000)	37.0 (35.0–41.0)	38.0 (35.0–41.0)	37.0 (34.0–41.0)	0.059
LVEDD, mm	45.0 (42.0–48.0)	45.0 (42.0–48.0)	45.0 (43.0–48.0)	45.0 (42.0–48.0)	0.810
LVESD, mm	30.0 (27.0–33.0)	30.0 (27.0–33.0)	30.0 (27.0–33.0)	30.0 (28.0–34.0)	0.001
LVEF, %	53.0 (42.0–60.0)	55.0 (46.0–60.0)	53.0 (43.0–60.0)	50.0 (39.0–55.0)	<0.001
Medications					
Aspirin	1,935 (95.8)	643 (95.5)	647 (96.1)	645 (95.7)	0.853
Statins	1,983 (98.2)	664 (98.7)	660 (98.1)	659 (97.8)	0.464
ACEI/ARB	1,300 (64.4)	470 (69.8)	439 (65.2)	391 (58.0)	<0.001
β-blockers	1,561 (77.3)	518 (77.0)	532 (79.0)	511 (75.8)	0.357
Outcomes, *n* (%)					
In-hospital death	92 (4.6)	7 (1.0)	19 (2.8)	66 (9.8)	<0.001
All-cause death	285 (14.1)	65 (9.7)	81 (12.0)	139 (20.6)	<0.001
Cardiac death	234 (11.6)	48 (7.1)	61 (9.1)	125 (18.5)	<0.001

ACEI, angiotensin-converting enzyme inhibitor; ARB, angiotensin II receptor blocker; CHF, chronic heart failure; CKD, chronic kidney disease; CRP, C-reactive protein; HBA1C, glycated hemoglobin; HDL, high-density lipoprotein; LDL, low-density lipoprotein; LAD, left atrial diameter; LVEDD, left ventricular end-diastolic diameter; LVEF, left ventricular ejection fraction; LVESD, left ventricular end-systolic diameter; PCI, percutaneous coronary intervention; SBP, systolic blood pressure; STEMI, ST-elevation myocardial infarction; TC, total cholesterol; TG, triglycerides; WBC, white blood cell.

The validation cohort included 1,443 AMI patients from the MIMIC-IV database, whose baseline characteristics are in Table S3. Compared to the discovery cohort, these patients were older, had more comorbidities, and had a higher 1-year all-cause mortality rate. Stratified analysis by NPNR tertiles showed similar results to the discovery cohort: the high NPNR group had the most systemic inflammation, poorest nutritional status, highest peak troponin T levels, and a higher 1-year all-cause mortality rate than the low NPNR group.

### Association of NPNR with myocardial injury and cardiac function

**Figure 2:**
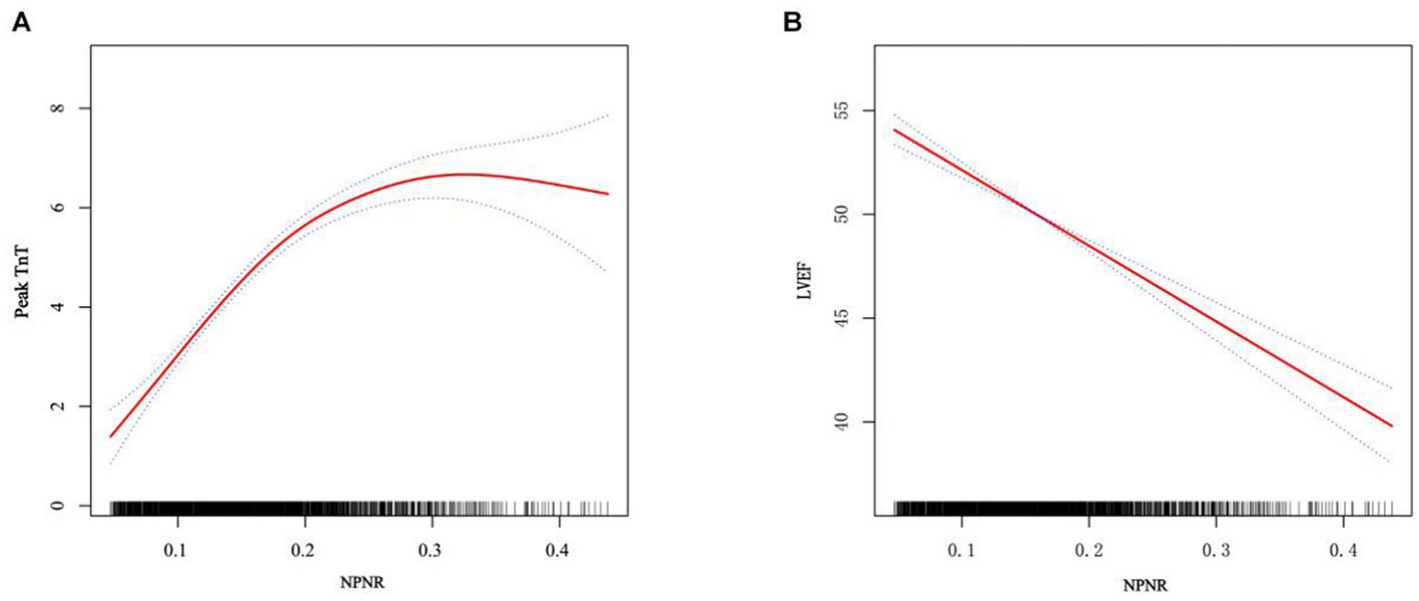
Generalized additive models (GAMs) showing the association between NPNR and **(A)** Peak TnT and **(B)** LVEF.

We used generalized additive models with smoothing curves to analyze the nonlinear relationships between NPNR and cardiac injury markers and function parameters. A significant positive nonlinear relationship was found between NPNR and Peak troponin T, with Peak troponin T levels rising as NPNR increased (Figure 2A). Conversely, a significant negative relationship was observed between NPNR and LVEF, where higher NPNR levels corresponded to lower LVEF (Figure 2B).

### Independent association between NPNR and mortality risks

**Figure 3:**
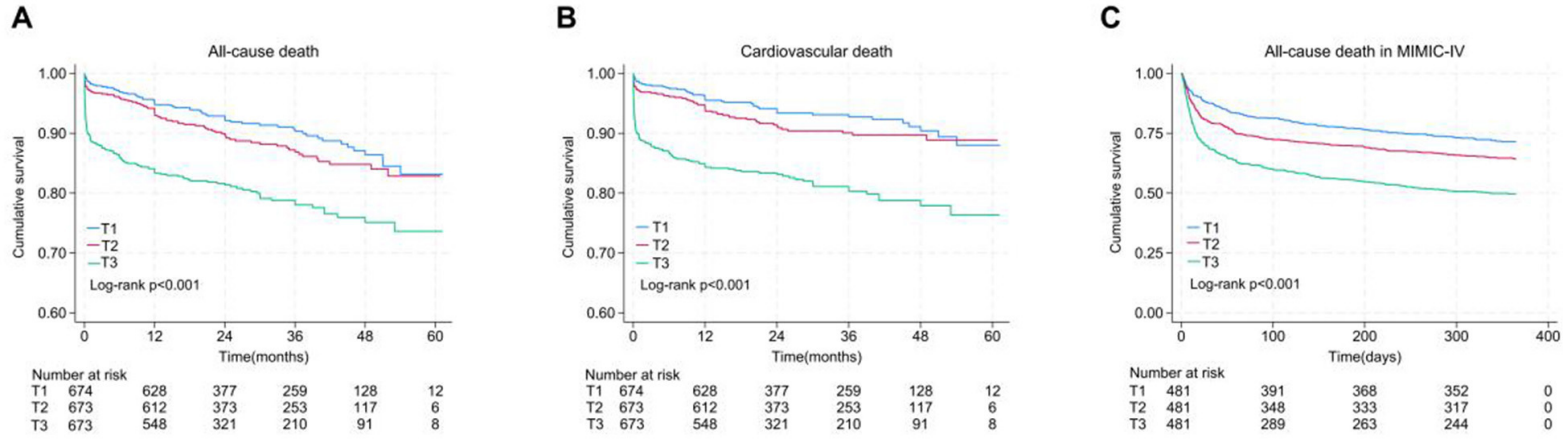
Kaplan–Meier curves for mortality outcomes. **(A)** All-cause mortality in the discovery cohort. **(B)** Cardiovascular mortality in the discovery cohort. **(C)** One-year all-cause mortality in the MIMIC-IV validation cohort.

Kaplan-Meier survival curves showed clear prognostic differences across NPNR tertiles in both cohorts. In the discovery cohort, survival rates varied significantly among the groups (Log-rank *P* < 0.001), with higher NPNR levels linked to increased risks of all-cause (Figure 3A) and cardiac mortality (Figure 3B), especially in T3. These results were validated in the MIMIC-IV cohort (Figure 3C), where despite a higher event rate, patients in the T3 had significantly lower 1-year survival compared to those in the T2 and T1 groups (Log-rank *P* < 0.001).

We employed multivariable Cox proportional hazards models to evaluate the prognostic significance of NPNR in relation to mortality. NPNR showed strong risk prediction in both unadjusted and age- and sex-adjusted models. Even in the fully adjusted model, NPNR was an independent risk factor for all-cause mortality (HR: 2.59, 95% CI: 1.21–5.54, *P* = 0.015) and cardiac mortality (HR: 2.87, 95% CI: 1.34–6.15, *P* = 0.007) as a continuous variable. Analysis by tertiles showed a dose-response relationship between NPNR and mortality risks. Patients in the T3 had a 2.19 times higher risk of all-cause mortality (HR: 2.19, 95% CI: 1.55–3.09, *P* < 0.001) and a 2.36 times higher risk of cardiac mortality (HR: 2.36, 95% CI: 1.60–3.48, *P* < 0.001) compared to those in the T1, with significant trends (*P* < 0.001; Table 2). This predictive value was confirmed in the MIMIC-IV cohort, where T3 had a 1.91 times higher risk of 1-year all-cause mortality than T1 (HR: 1.91, 95% CI: 1.51–2.43, *P* < 0.001; Table 3).

**Table 2:** Multivariate cox regression analyses for the association between NPNR and all-cause and cardiac mortality.

Variable	Model 1		Model 2		Model 3	
	HR (95%CI)	*p*-value	HR (95%CI)	*p*-value	HR (95%CI)	*p*-value
All-cause death						
NPNR	2.54 (1.54–4.19)	<0.001	4.42 (2.57–7.58)	<0.001	2.59 (1.21–5.54)	0.015
NPNR tertiles						
T1	1.00 (Ref)		1.00 (Ref)		1.00 (Ref)	
T2	1.28 (0.92–1.77)	0.143	1.49 (1.08–2.07)	0.016	1.31 (0.93–1.84)	0.119
T3	2.42 (1.80–3.25)	<0.001	2.89 (2.15–3.89)	<0.001	2.19 (1.55–3.09)	<0.001
*P* for trend		0.001		<0.001		<0.001
Cardiac death						
NPNR	2.92 (1.78–4.78)	<0.001	5.01 (2.94–8.52)	<0.001	2.87 (1.34–6.15)	0.007
NPNR tertiles						
T1	1.00 (Ref)		1.00 (Ref)		1.00 (Ref)	
T2	1.30 (0.89–1.90)	0.175	1.49 (1.02–2.18)	0.038	1.29 (0.87–1.90)	0.211
T3	2.91 (2.09–4.06)	<0.001	3.45 (2.47–4.82)	<0.001	2.36 (1.60–3.48)	<0.001
*P* for trend		<0.001		<0.001		<0.001

Model 1: unadjusted

Model 2: adjusted for age and gender

Model 3: adjusted for age, gender, current smoking, hypertension, diabetes, CKD, CHF, STEMI, heart rate, SBP, LVEF, PCI, creatinine, CRP, peak TnT, TC, HDL-C, aspirin, ACEI/ARBs, β-blockers and statins.

**Table 3:** Multivariable cox regression analysis of the association between NPNR and 1-year all-cause mortality in MIMCI-IV cohort.

Variable	Model 1		Model 2		Model 3	
	HR (95%CI)	*p*-value	HR (95%CI)	*p*-value	HR (95%CI)	*p*-value
All-cause death						
NPNR	6.02 (3.80–9.54)	<0.001	7.82 (4.81–12.72)	<0.001	4.11 (2.37–7.13)	<0.001
NPNR tertiles						
T1	1.00 (Ref)		1.00 (Ref)		1.00 (Ref)	
T2	1.34 (1.07–1.68)	0.010	1.30 (1.04–1.63)	0.020	1.24 (0.96–1.59)	0.100
T3	2.15 (1.74–2.65)	<0.001	2.16 (1.75–2.67)	<0.001	1.91 (1.51–2.43)	<0.001
*P* for trend		<0.001		<0.001		<0.001

Model 1: unadjusted

Model 2: adjusted for age and gender

Model 3: adjusted for age, gender, white, hypertension, diabetes, dyslipidemia, CKD, CHF, heart rate, SBP, PCI, creatinine, peak TnT, aspirin, ACEI/ARBs, β-blockers and statins.

**Figure 4:**
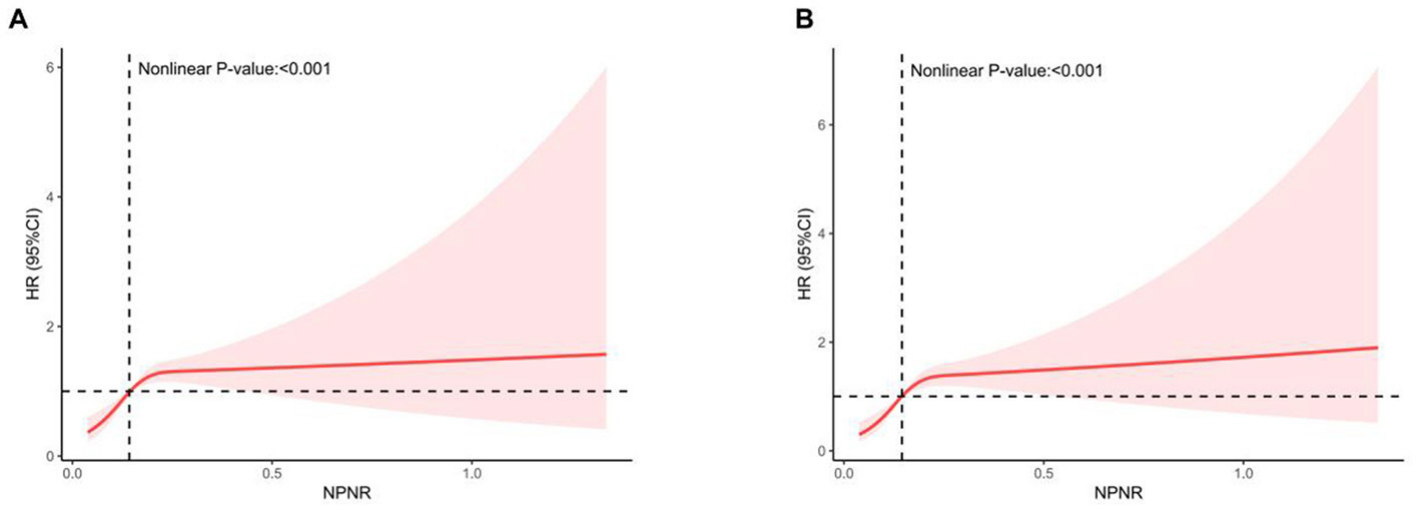
Restricted cubic spline (RCS) curve of the NPNR and death in patients with AMI. **(A)** RCS curve for all-cause mortality. **(B)** RCS curve for cardiovascular mortality.

We used restricted cubic spline models to analyze the relationship between NPNR and mortality risks. In the discovery cohort, a significant J-shaped, non-linear association was found between NPNR and both all-cause (Figure 4A) and cardiac mortality (Figure 4B) after adjusting for confounders (*P* for nonlinearity < 0.001). However, in the MIMIC-IV validation cohort, the association with 1-year all-cause mortality was linear (*P* for nonlinearity = 0.6779; Figure S2).

### Predictive performance and incremental prognostic value of NPNR

ROC curve analysis assessed the predictive values of NPNR and other inflammation-related indices (NLR, SII, and PNI) for mortality. For all-cause mortality, NPNR showed reliable predictive ability (AUC = 0.621) and was statistically similar to NLR (AUC = 0.630) and SII (AUC = 0.608), but superior to PNI (AUC = 0.574). For cardiovascular mortality, all indices improved, with NPNR maintaining predictive value (AUC = 0.652), comparable to NLR (AUC = 0.658) and SII (AUC = 0.634), and significantly better than PNI (AUC = 0.589; Figure S3). The differences were not statistically significant. To evaluate the incremental prognostic value of NPNR, we constructed a baseline clinical model. The baseline C-statistic was 0.807 (95% CI: 0.783–0.830). The addition of NPNR to the baseline model yielded a significant improvement in overall discrimination (C-statistic increased to 0.811, 95% CI: 0.787–0.834; *P* = 0.011). Furthermore, reclassification analysis confirmed that the inclusion of NPNR significantly improved risk stratification, with a continuous net reclassification improvement (NRI) of 0.368 (95% CI: 0.166–0.455; *P* = 0.013) and an integrated discrimination improvement (IDI) of 0.011 (95% CI: 0.003–0.020; *P* = 0.013; Table 4).

**Table 4:** The incremental prognostic ability of NPNR for all-cause mortality.

Index	Base model	Base model + NPNR	*P*
C-statistic (95% CI)	0.807 (0.783–0.830)	0.811 (0.787–0.834)	0.011
Continuous NRI (95% CI)	Ref	0.368 (0.166–0.455)	0.013
IDI (95% CI)	Ref	0.011 (0.003–0.020)	0.013

IDI, integrated discrimination improvement; NRI, net reclassification improvement.

### Subgroup analysis and sensitivity analysis

**Figure 5:**
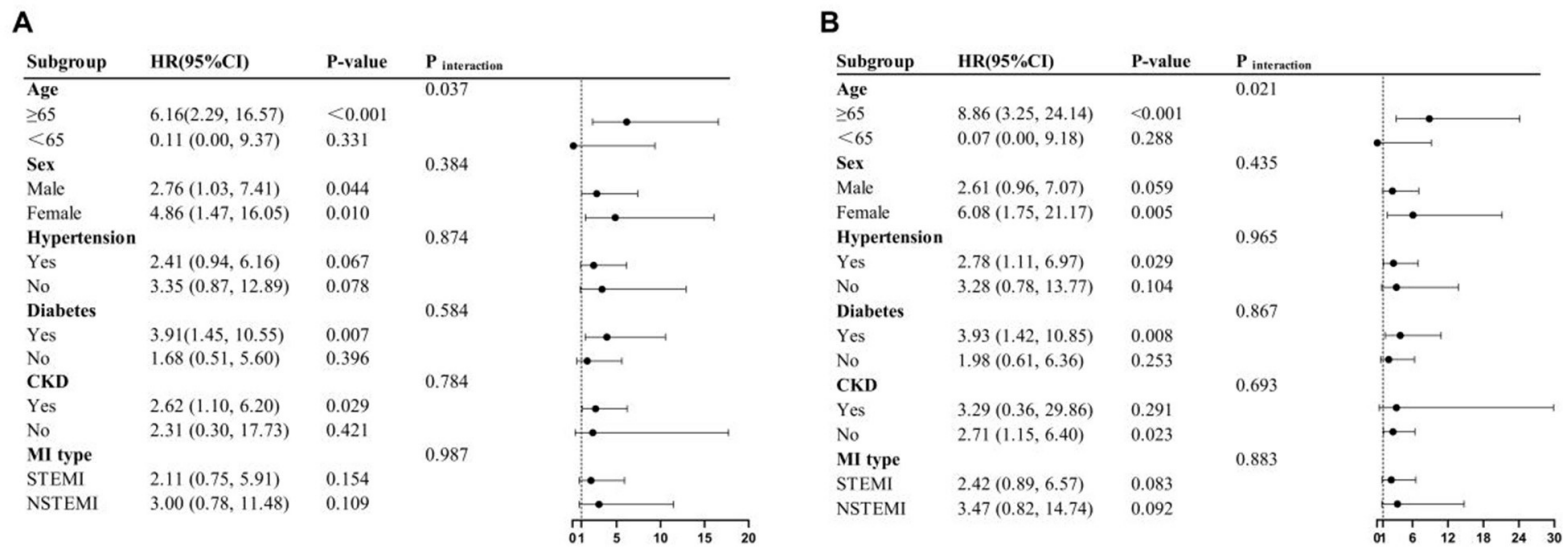
Forest plots of subgroup analyses for all-cause mortality **(A)** and cardiovascular mortality **(B)**.

We conducted stratified analyses to assess the robustness of the NPNR association with adverse prognosis across various factors like age, sex, hypertension, diabetes, CKD, and myocardial infarction type. The predictive value of NPNR for all-cause (Figure 5A) and cardiac mortality (Figure 5B) was consistent across most subgroups, with no significant interaction effects from sex, hypertension, diabetes, CKD, or myocardial infarction type. However, age significantly influenced the predictive efficacy of NPNR (*P* for interaction < 0.037). In patients aged 65 and older, elevated NPNR was strongly associated with increased risks of all-cause (HR: 6.16, 95% CI: 2.29–16.57, *P* < 0.001) and cardiac mortality (HR: 8.86, 95% CI: 3.25–24.14, *P* < 0.001), while this association was not statistically significant in younger patients.

To ensure comparability with the MIMIC-IV validation cohort's primary endpoint of 1-year all-cause mortality, we truncated the discovery cohort's follow-up to 1 year (Table S4). The Cox regression analysis remained consistent, showing that NPNR was an independent predictor of 1-year mortality (HR: 3.66, 95% CI: 1.73–7.74, *P* < 0.001). In the fully adjusted model, patients in the T3 had a 2.90 times higher risk of 1-year mortality compared to those in the T1 (HR: 2.90, 95% CI: 1.87–4.49, *P* < 0.001).In including patients with severe inflammatory responses (WBC ≥20 × 1 × 10^9^/L or CRP ≥200 mg/L), higher NPNR remained robustly and independently associated with an increased risk of all-cause mortality (Model 3, T3 vs. T1: HR 1.70, 95% CI 1.18–2.46, *P* = 0.004; Table S5).

## Discussion

The prognostic value of biomarkers combining inflammation and nutrition in AMI is under-researched. This study evaluated the NPNR in 2020 AMI patients and validated it in 1,443 others. Patients categorized into higher NPNR tertiles demonstrated progressively deteriorating clinical profiles, marked by elevated inflammatory markers, diminished cardiac function, and increased mortality rates. Multivariable analysis revealed that NPNR remained an independent predictor of both all-cause and cardiovascular mortality after controlling for potential confounders, which were successfully validated in the MIMIC-IV cohort.

It is worth noting that while the addition of NPNR significantly enhanced the predictive model, the absolute increment in the C-statistic was relatively modest (from 0.807 to 0.811). This reflects a well-documented statistical phenomenon: when a baseline model already exhibits strong discriminative ability (C-statistic > 0.80), the C-statistic becomes highly insensitive to the introduction of novel predictors (18). Consequently, the clinical relevance of this absolute increment should be interpreted cautiously. To address this limitation, we utilized the Net Reclassification Improvement (NRI) and Integrated Discrimination Improvement (IDI) to better capture shifts in individual risk profiling. The substantial continuous NRI of 0.368 observed in our study indicates that NPNR facilitates clinically meaningful risk reclassification for a considerable proportion of patients. This demonstrates its practical value in refining individualized risk stratification beyond standard clinical variables.

Prior research has demonstrated the predictive utility of combined inflammatory-nutritional indices. Huang et al. showed neutrophil-to-HDL ratio outperformed traditional markers in elderly AMI patients (19). Our prior analysis of 2,111 AMI patients revealed systemic immune-inflammation index (SII) independently predicted mortality, especially in diabetic subgroups (17). However, Basta et al. found CONUT score predicted mortality in elderly STEMI patients while PNI alone did not (20). Neutrophils reflect established acute coronary syndrome (ACS) pathophysiology. Generalized additive model analysis revealed a significant nonlinear direct association between NPNR and peak troponin T, contrasted by a significant inverse relationship with LVEF, indicating direct relationship between inflammatory burden and myocardial injury severity. As key innate immune cells, neutrophils play central role in CAD inflammatory cascade (21). During early AMI, neutrophil activation with myeloperoxidase depletion promotes platelet-neutrophil aggregates and occlusive thrombosis (22). Evidence from Karabinos et al. highlights admission neutrophil counts as an independent prognostic marker for in-hospital adverse events in ACS (23). Consistent with this, the neutrophil-to-lymphocyte ratio (NLR) is frequently linked to negative cardiovascular results. Notably, analysis of the NHANES dataset identified elevated NLR as an independent contributor to coronary heart disease risk, especially among specific subgroups such as women, older individuals, and patients with multiple chronic conditions (24). Agarwal et al emphasized the prognostic value of NLR across all coronary heart disease (CHD) stages, with elevated levels linked to larger infarct sizes and adverse cardiac remodeling post-MI (25).

PNI captures nutritional-immune reserve through serum albumin and lymphocyte count, holding independent prognostic value in cardiovascular disease. Our data show T3 group patients had highest neutrophil counts and lowest albumin levels, indicating severe nutritional-immune impairment. Accumulating evidence confirmed the robust prognostic capacity of PNI in coronary heart disease and ACS patients. Zhang et al.'s (26) meta-analysis revealed low PNI predicts higher long-term mortality and MACE risk in CHD patients (26). Chang et al. (27) provided further validation regarding the prognostic value of PNI for long-term survival in ACS patients treated with PCI (27). Malnutrition prevalence and prognostic significance in ACS are well-established. Raposeiras Roubín et al. (28) demonstrated malnutrition's association with adverse outcomes in ACS (28), while Kang et al. (29) showed high prevalence and prognostic implications in PCI-treated ACS patients (29). PNI can serve as both an independent marker and be combined with other biomarkers. Sun et al. confirmed PNI-HbA1c combination better predicts prognosis in diabetic ACS patients (30). Özkan et al. (31) found PNI correlates with coronary stenosis severity (31), and Ma et al. (32) demonstrated PNI's association with NYHA class in elderly CHD patients (32). Meanwhile, the risk of developing contrast-induced nephropathy in the ACS population can be assessed using the PNI (33, 34). Unlike studies restricted to specific phenotypes (elderly STEMI), our inclusion of diverse AMI phenotypes (STEMI and NSTEMI) enhances generalizability to real-world high-risk populations (20).

RCS analysis revealed a J-shaped NPNR-mortality association in the discovery cohort, but a linear relationship in MIMIC-IV, suggesting a potential NPNR “threshold effect”. Below this threshold, mild inflammatory-nutritional states may facilitate myocardial repair, whereas crossing it triggers detrimental imbalances that sharply elevate mortality (35). Conversely, MIMIC-IV patients were older and critically ill with heavier comorbidities; their higher baseline NPNR indicates they likely already exceeded this physiological threshold, yielding a continuous linear risk. Although treatment modalities (e.g., PCI rates) differ significantly between the discovery and validation cohorts, NPNR's prognostic value transcends specific mechanical interventions. As a universal surrogate for systemic vulnerability, it exhibits remarkable applicability in heterogeneous real-world environments. Stratified analyses revealed a significant age interaction. NPNR demonstrated robust risk stratification capacity in patients ≥65 years. This highlights that elderly patients commonly experience immunosenescence and malnutrition, with reduced metabolic reserve and impaired myocardial repair capacity, rendering them less tolerant to inflammation-nutrition imbalance and thereby amplifying adverse effect (36–38). Our results imply that the NPNR serves as an integrated biomarker that accurately reflects the exacerbated vulnerability to acute inflammation and nutritional depletion in the aging AMI population.

The applicability of inflammation-nutrition indices extends beyond cardiovascular diseases, although variations contingent on specific clinical contexts are observed. In surgical and oncology populations, indices such as BMI/PNI and postoperative NLR have been effective in predicting complications, including pancreatic fistula (39) and anastomotic leakage (40), while high PNI levels have been associated with improved survival in colorectal cancer patients (41). Nonetheless, inconsistencies in prognostic trends have been documented. In the septic population, Cai et al. (15) reported that the link between NPNR and death followed a V-shaped pattern (15), which contrasts with the nonlinear risk increase observed in our AMI cohort. This discrepancy likely reflects differing pathophysiological mechanisms: primary systemic inflammation in sepsis vs. cardiac injury-induced secondary inflammation in AMI.

Elevated neutrophils drive myocardial injury *via* reactive oxygen species and cytokine release, with neutrophil-derived extracellular vesicles further exacerbating microvascular obstruction (42). Concurrently, reduced PNI indicates immune-nutritional depletion, impairing myocardial repair and increasing cachexia risk (43). Supporting this dual vulnerability, Borda et al. (44) found that higher NLR correlated with worse functional outcomes in older adults (44). Beyond neutrophil quantification, the inflammatory microenvironment in ACS is characterized by complex cytokine dysregulation. A comparison by Maaniitty et al. (45) revealed that cytokine profiles in the circulation vary significantly when distinguishing ACS from stable CAD. The study highlighted an upregulation of pro-inflammatory cytokines, notably interleukin-6 and macrophage colony-stimulating factor, in the ACS cohort (45). This dynamic inflammatory milieu is further modulated by the balance between pro- and anti-inflammatory cytokines, which in turn influences platelet aggregability and thrombotic risk in ACS patients (46). Crucially, NPNRcaptures the “inflammation-nutrition vicious cycle” governing AMI prognosis. Hyperactive neutrophils release pro-inflammatory cytokines that directly suppress hepatic albumin synthesis and induce lymphocyte apoptosis, rapidly driving down PNI (47). Conversely, the resulting hypoalbuminemia and lymphopenia impair antioxidant defenses and immune regulation. During acute ischemia-reperfusion injury, this unbuffered inflammatory burst exacerbates myocardial damage and extracellular matrix degradation (48). Ultimately, this unresolved inflammatory-nutritional deficit disrupts tissue healing, significantly accelerating adverse ventricular remodeling and increasing long-term mortality risk.

This study has several limitations. Primarily, the retrospective observational design establishes an association, not causation, between NPNR and mortality. Moreover, NPNR was measured only at admission, potentially missing dynamic changes in inflammation and nutrition during AMI. Future research should elucidate how longitudinal NPNR trajectories correlate with clinical outcomes. Furthermore, the discrepancy in tertile-based cut-offs across the dual Chinese and US cohorts limits the direct clinical applicability. Therefore, large-scale, prospective studies are urgently needed to establish an optimal and standardized clinical threshold for routine practice. Compounding this database reliance, outcomes in the validation cohort were retrieved directly from the database, lacking precise adjudication of specific causes of death. Lastly, the unavailability of critical clinical variables—such as the Killip class and GRACE score—coupled with the exclusion of patients with incomplete records, may introduce residual confounding and selection bias.

## Conclusion

In conclusion, this retrospective dual-cohort study suggests that NPNR may serves a novel and potential independent predictor of long-term all-cause and cardiovascular mortality in patients with AMI. The prognostic value of NPNR was consistent across both Chinese and US populations, suggesting its potential generalizability. Given its accessibility, cost-effectiveness, and predictive power, NPNR represents a valuable addition to current risk stratification strategies, potentially guiding more personalized management for high-risk AMI patients, with this predictive value being especially pronounced in elderly patients. Future large-scale, prospective studies are warranted to validate its true clinical utility.

## Data Availability

The original contributions presented in the study are included in the article/Supplementary material, further inquiries can be directed to the corresponding authors.
