# Supplementary material for: Association of neutrophil-to-prognostic nutritional index ratio with long-term mortality in acute myocardial infarction from Chinese and US cohorts
Source: Front Nutr. 2026 Jun 18;13:1814469. doi: 10.3389/fnut.2026.1814469 (PMC13326627; doi:10.3389/fnut.2026.1814469)
Supplement: Supplementary file 1 [file Supplementary_file_1.docx]

**Table S1 Collinearity diagnostics for variables included in the multivariable models**

| Variable | VIF | Tolerance (1/VIF) |
| --- | --- | --- |
| LVEF | 2.77 | 0.361 |
| CHF | 2.56 | 0.391 |
| Age | 1.57 | 0.637 |
| Peak TnT | 1.50 | 0.666 |
| STEMI | 1.43 | 0.698 |
| Male | 1.39 | 0.718 |
| Current smoking | 1.37 | 0.730 |
| CKD | 1.31 | 0.763 |
| Creatinine | 1.30 | 0.770 |
| TC | 1.28 | 0.784 |
| PCI | 1.23 | 0.812 |
| HDL | 1.20 | 0.830 |
| Heart Rate | 1.19 | 0.838 |
| SBP | 1.15 | 0.866 |
| CRP | 1.15 | 0.873 |
| NPNR | 1.09 | 0.917 |
| Hypertension | 1.08 | 0.922 |
| Diabetes | 1.07 | 0.937 |
| Mean VIF | 1.41 |  |

Abbreviations: VIF, variance inflation factor.

**Table S2 Univariable analysis for the all-cause mortality**

| Variable | HR (95%CI) | P-value |
| --- | --- | --- |
| Demographics |  |  |
| Age, years | 1.09 (1.07–1.10) | <0.001 |
| Male, n (%) | 0.53 (0.42–0.68) | <0.001 |
| Current Smoker, n (%) | 0.40 (0.31–0.52) | <0.001 |
| Heart Rate, bpm | 1.02 (1.02–1.03) | <0.001 |
| SBP, mmHg | 0.99 (0.99–1.00) | 0.038 |
| STEMI | 0.81 (0.64–1.03) | 0.082 |
| PCI | 0.25 (0.20–0.31) | <0.001 |
| Comorbidities, n (%) |  |  |
| Hypertension | 1.29 (1.01–1.66) | 0.045 |
| Diabetes | 1.53 (1.21–1.93) | <0.001 |
| CKD | 3.85 (2.94–5.04) | <0.001 |
| CHF | 3.17 (2.50–4.02) | <0.001 |
| Laboratory data |  |  |
| Creatinine, μmol/L | 1.00 (1.00–1.00) | <0.001 |
| CRP, mg/L | 1.01 (1.01–1.01) | <0.001 |
| Peak TnT, ng/mL | 1.02 (0.99–1.05) | 0.222 |
| TC, mmol/L | 0.92 (0.82–1.04) | 0.172 |
| HDL, mmol/L | 1.17 (0.78–1.77) | 0.446 |
| Echocardiography |  |  |
| LVEF, % | 0.95 (0.94– 0.96) | <0.001 |
| Medications |  |  |
| Aspirin | 0.33 (0.22– 0.48) | <0.001 |
| ACEI/ARB | 0.69 (0.54–0.87) | 0.002 |
| β-blockers | 0.57 (0.44– 0.72) | <0.001 |
| Statins | 0.14(0.09– 0.21) | <0.001 |

ACEI: Angiotensin-converting enzyme inhibitor; ARB: Angiotensin II receptor blocker; CHF: Chronic heart failure; CKD: Chronic kidney disease; CRP: C-reactive protein; HDL: High-density lipoprotein; LDL: Low-density lipoprotein; LAD: Left atrial diameter; PCI: Percutaneous coronary intervention; SBP: Systolic blood pressure; STEMI: ST-elevation myocardial infarction; TC: Total cholesterol.

**Table S3 Baseline characteristics of the study population in MIMIC-IV cohort**

| Characteristics | Total  (N=1443) | T1  (n=481) | T2  (n=481) | T3  (n=481) | P-value |
| --- | --- | --- | --- | --- | --- |
| Demographics |  |  |  |  |  |
| Age, years | 71.6(62.5-81.1) | 70.5(60.8-80.5) | 72.3(63.5-81.6) | 71.9(64.0-81.0) | 0.183 |
| Male, n (%) | 875 (60.6%) | 287 (59.7%) | 305 (63.4%) | 283 (58.8%) | 0.302 |
| White race, n (%) | 839 (58.1%) | 265 (55.1%) | 289 (60.1%) | 285 (59.3%) | 0.244 |
| Heart rate, bpm | 86.0 (75.0-101.0) | 85.0 (75.0-98.0) | 87.0 (74.0-101.0) | 88.0 (75.0-104.0) | 0.044 |
| SBP, mmHg | 121 (106-141) | 122 (107-142) | 121 (107-140) | 120 (103-140) | 0.377 |
| PCI | 488 (33.8%) | 168 (34.9%) | 172 (35.8%) | 148 (30.8%) | 0.215 |
| Comorbidities, n (%) |  |  |  |  |  |
| Hypertension | 532 (36.9%) | 179 (37.2%) | 172 (35.8%) | 181 (37.6%) | 0.819 |
| Diabetes | 632 (43.8%) | 216 (44.9%) | 209 (43.5%) | 207 (43.0%) | 0.828 |
| Dyslipidemia | 998 (69.2%) | 347 (72.1%) | 349 (72.6%) | 302 (62.8%) | 0.001 |
| CKD | 484 (33.5%) | 160 (33.3%) | 162 (33.7%) | 162 (33.7%) | 0.988 |
| CHF | 948 (65.7%) | 310 (64.4%) | 324 (67.4%) | 314 (65.3%) | 0.619 |
| Laboratory data |  |  |  |  |  |
| WBC, ×10⁹/L | 11.1 (8.2-14.3) | 7.5 (5.6-9.8) | 11.2 (9.7-13.1) | 14.6 (12.3-17.1) | <0.001 |
| Neutrophil, ×10⁹/L | 9.0(6.0-11.9) | 4.92(3.4-6.2) | 9.2(8.0-10.4) | 13.5 (11.4-15.6) | <0.001 |
| Lymphocyte, ×10⁹/L | 1.1(0.7-1.5) | 1.1(0.6-1.6) | 1.1(0.7-1.6) | 1.0(0.6-1.3) | <0.001 |
| Hemoglobin, g/dL | 10.7 (8.9-12.4) | 10.5 (8.8-12.0) | 11.0 (8.9-12.9) | 10.7 (9.0-12.2) | 0.021 |
| Platelet, ×10⁹/L | 203 (146-261) | 179 (124-231) | 199 (153-260) | 229 (171-286) | <0.001 |
| Albumin, g/L | 36.0 (31.0-41.0) | 39.0 (34.0-42.0) | 37.0 (33.0-41.0) | 32.0 (28.0-37.0) | <0.001 |
| HbA1c, % | 5.9 (5.5-7.1) | 5.9 (5.5-7.0) | 6.0 (5.5-7.2) | 5.9 (5.5-6.9) | 0.856 |
| Creatinine, μmol/L | 106 (71-168) | 97 (71-159) | 106 (71-159) | 115 (80-186) | 0.001 |
| Peak TnT, ng/mL | 0.82(0.2-2.9) | 0.6(0.2-1.8) | 0.9(0.3-3.2) | 1.1(0.3-3.5) | <0.001 |
| Medications |  |  |  |  |  |
| Aspirin | 1129 (78.2%) | 353 (73.4%) | 382 (79.4%) | 394 (81.9%) | 0.004 |
| Statins | 1062 (73.6%) | 334 (69.4%) | 375 (78.0%) | 353 (73.4%) | 0.011 |
| ACEI/ARB | 668 (46.3%) | 227 (47.2%) | 222 (46.2%) | 219 (45.5%) | 0.872 |
| β-blockers | 1147 (79.5%) | 372 (77.3%) | 390 (81.1%) | 385 (80.0%) | 0.333 |
| Outcomes, n (%) |  |  |  |  |  |
| 365-day mortality | 551 (38.2%) | 137 (28.5%) | 172 (35.8%) | 242 (50.3%) | <0.001 |

|  |
| --- |

Abbreviations: ACEI, angiotensin-converting enzyme inhibitor; ARB, angiotensin II receptor blocker; CKD, chronic kidney disease; CHF, chronic heart failure; HbA1c, glycated hemoglobin; PCI, percutaneous coronary intervention; SBP, systolic blood pressure;WBC:White blood cell..

**Table S4 Multivariable Cox regression analysis of the association between NPNR and 365-day all-cause mortality in the discovery cohort**

| Variable | Model 1 |  | Model 2 |  | Model 3 |  |
| --- | --- | --- | --- | --- | --- | --- |
|  | HR (95%CI) | P-value | HR (95%CI) | P-value | HR (95%CI) | P-value |
| All-cause death |  |  |  |  |  |  |
| NPNR | 3.12 (1.88–5.16) | <0.001 | 5.89 (3.39–10.22) | <0.001 | 3.66 (1.73–7.74) | <0.001 |
| NPNR tertiles |  |  |  |  |  |  |
| T1 | 1.00 (Ref) |  | 1.00 (Ref) |  | 1.00 (Ref) |  |
| T2 | 1.24 (0.80–1.95) | 0.336 | 1.38 (0.88–2.16) | 0.158 | 1.19 (0.75–1.89) | 0.464 |
| T3 | 3.44 (2.35–5.03) | <0.001 | 4.01 (2.74–5.87) | <0.001 | 2.90 (1.87–4.49) | <0.001 |
| P for trend |  | <0.001 |  | <0.001 |  | <0.001 |

Model 1: Unadjusted.

Model 2: Adjusted for age and gender.

Model 3: Adjusted for age, gender, current smoking, hypertension, diabetes, CKD, CHF, STEMI, heart rate, SBP, LVEF, PCI, creatinine, CRP, peak TnT, TC, HDL-C, aspirin, ACEI/ARBs,β-blockers and statins.

**Table S5 Sensitivity analysis of the association between NPNR and all-cause mortality in the discovery cohort after including patients with a severe inflammatory response**

| Variable | Model 1 |  | Model 2 |  | Model 3 |  |
| --- | --- | --- | --- | --- | --- | --- |
|  | HR (95%CI) | P-value | HR (95%CI) | P-value | HR (95%CI) | P-value |
| All-cause death |  |  |  |  |  |  |
| NPNR | 1.39 (1.27–1.53) | <0.001 | 1.53 (1.37–1.71) | <0.001 | 1.26 (1.03–1.55) | 0.023 |
| NPNR tertiles |  |  |  |  |  |  |
| T1 | 1.00 (Ref) |  | 1.00 (Ref) |  | 1.00 (Ref) |  |
| T2 | 1.52 (1.11–2.08) | 0.009 | 1.54 (1.12–2.10) | 0.007 | 1.16 (0.80–1.67) | 0.426 |
| T3 | 2.78 (2.09–3.71) | <0.001 | 2.66 (1.99–3.55) | <0.001 | 1.70 (1.18–2.46) | 0.004 |
| P for trend |  | <0.001 |  | <0.001 |  | 0.003 |

Model 1: Unadjusted.

Model 2: Adjusted for age and gender.

Model 3: Adjusted for age, gender, current smoking, hypertension, diabetes, CKD, CHF, STEMI, heart rate, SBP, LVEF, PCI, creatinine, CRP, peak TnT, TC, HDL-C, aspirin, ACEI/ARBs,β-blockers and statins.


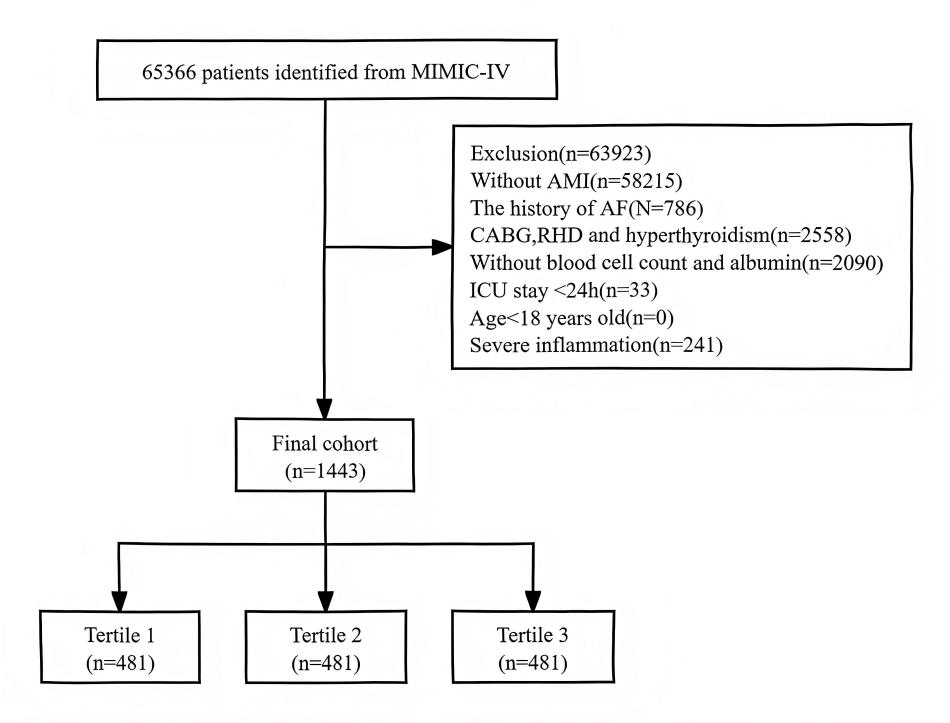


FIGURE S1 Flowchart of patient selection in MIMIC-IV validation cohort.


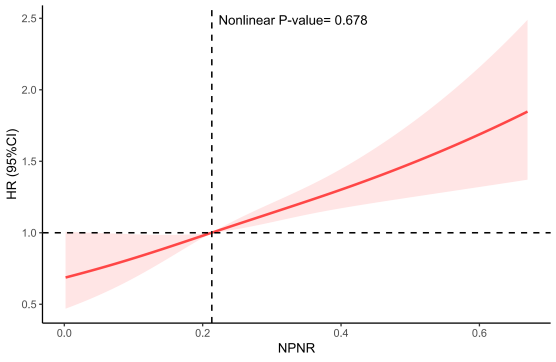


FIGURE S2 Restricted cubic spline curve of the NPNR and all-cause mortality in the MIMIC-IV validation cohort.


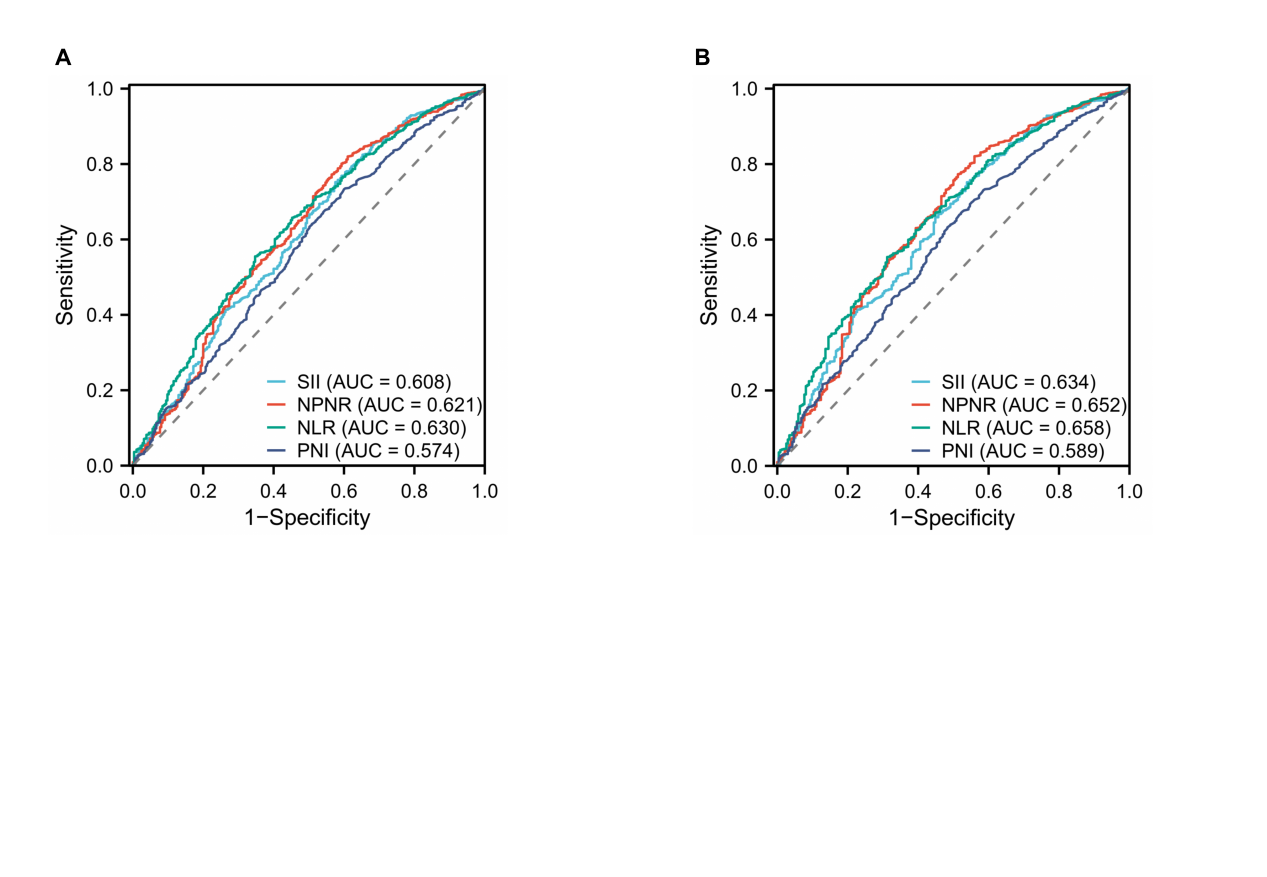


FIGURE S3 ROC curves for for all-cause mortality (A) and cardiovascular mortality (B).
